# Hepatitis B virus infection in hilly/mountainous regions of southeastern China: a locality-dependent epidemiology

**DOI:** 10.1186/s12879-017-2922-7

**Published:** 2017-12-29

**Authors:** Ping Chen, Qinfen Xie, Ting Chen, Jiawei Wu, Jie Wu, Bing Ruan, Zhiqin Zhang, Hainv Gao, Lanjuan Li

**Affiliations:** 1 Shulan (Hangzhou) Hospital, Zhejiang University International Hospital, Hangzhou, 310012 China; 2 State Key Laboratory for Diagnosis and Treatment of Infectious Diseases, Collaborative Innovation Center for Diagnosis and Treatment of Infectious Diseases, The First Affiliated Hospital, College of Medicine, Zhejiang University, 79 Qing-Chun Road, Hangzhou, 310003 China; 3 College of Medicine, Zhejiang University, Hangzhou, 310058 China; 4 Xianju Hospital of Traditional Chinese Medicine, Zhenjiang, Xianju 317300 China

**Keywords:** Hepatitis B virus, Community-based epidemiological study, Expanded program of immunization, Continuous HBV transmission

## Abstract

**Background:**

The overall prevalence of hepatitis B virus (HBV) infection in China is declining. The purpose of this study was to use a community-based epidemiological study to update the infection status of hepatitis B virus (HBV) in mountainous regions of China, and to evaluate the impact of the Expanded Program of Immunization (EPI) on HBV transmission.

**Methods:**

In total, 10,383 participants were selected by multi-stage stratified random cluster sampling in two mountainous regions, Xianju and Anji, in Zhejiang province, China.

**Results:**

The positive rates of hepatitis B virus surface antigen (HBsAg), anti-HBV core antigen (anti-HBc), and anti-HBV surface antigen (anti-HBs) were 9.5%, 33.9%, and 51.0%, respectively. Positive HBV markers were more frequently detected in males than in females (*P* < 0.01). The alanine aminotransferase (ALT) levels were elevated (>38 IU/L) in 15.3% of the HBsAg-positive and 6.3% of the HBsAg-negative subjects. The α-fetoprotein (AFP) level was elevated in 0.8% of the HBsAg-positive participants who were older than 30 years old.

**Conclusions:**

The epidemiology of HBV infection is location dependent. The prevalence of HBV infection in the mountainous regions is higher than the national levels. Moreover, HBV infection in women of childbearing age is up to 10%, which represents a main factor for continuous HBV transmission.

## Background

Hepatitis B virus (HBV) infection is one of the most common viral infections [1]. Globally, almost two billion people have been exposed to HBV infection at some stage of their lives, and an estimated 240 million people are chronically infected. Approximately 780,000 patients die from advanced liver diseases caused by chronic HBV infection each year [2–4].

China has a high prevalence of chronic HBV infection. However, there is a significant difference in the HBV infection rate among different regions in China. Generally speaking, the prevalence of HBV infection in eastern, coastal, or rural areas is higher than that in western, inland, or urban regions [5]. In 1992, China released the first national hepatitis seroepidemiological survey, which showed that the prevalence of hepatitis B virus surface antigen (HBsAg) in the general population aged 1–59 years old was 9.75% [6]. The vertical infection of HBV from a HBV-positive mother to her infant is a major route for HBV transmission. To control HBV transmission, the Expanded Program of Immunization (EPI) for newborns was established in China in 1992. This program has been a great success and has resulted in a decline in the prevalence of chronic HBV infection, from 9.75% in 1992 to 7.18% in 2006 [7]. The overall prevalence of chronic HBV carriers in Zhejiang province was approximately 8.79% in 2006, a decline from 11.61% in 1992 [8].

In hilly/mountainous areas of China, the prevalence of chronic HBV infection in the general population was 15.94% in 1993, [9, 10]. Mountains and hills account for 70.4% of the total land area of Zhejiang province, which is the largest of all Chinese provinces. An epidemiological study conducted in 2007 found that the prevalence of chronic HBV infection was 10.19% in the mountainous areas of Zhejiang province [11], which had declined from 15.94%, but it was still much higher than the provincial rate of 6.75% in Zhejiang province [12].

HBV seroepidemiological studies in the hilly/mountainous areas of China are relatively rare. The current status of chronic HBV infection and the related risk factors in those areas need to be assessed. The hilly/mountainous area in Xianju and Anji accounts for 80% and 70% of the territory, respectively. Geographically, the two counties are separately located. The economic development level also differs between the two counties. Therefore, we conducted a community-based epidemiological study in two representative hilly/mountainous areas in southeastern China to determine the current chronic HBV infection rate in the general population, to study the risk factors, and to evaluate the impact of the EPI on HBV transmission.

## Methods

### Study population

Under the sponsorship of the Mega-Project for National Science and Technology Development for the “12th Five-Year Plan of China” and the Department of Health of Zhejiang province, we initiated this HBV infection epidemiological study in two major hilly counties of Zhejiang province. This study was conducted between October 2012 and March 2014.

### Sample size

Based on the previous HBV epidemiological studies, we assumed that the incidence of HBV infection in hilly/mountainous regions would be about 5–10%. Sample size was estimated with the following formula:

$$ \mathrm{n}=\frac{{\mathrm{Z}}_{\upalpha}^2\mathrm{P}\left(1\hbox{-} \mathrm{P}\right)}{\updelta^2} $$

The calculated sample size is 8760 when we assume 80% power, 95% confidence level, and the incidence of HBV is set at 5%. In our analysis, we also take into account 20% possible attrition of subjects, and the final sample size is 9855.

### Sampling strategy

Our study populations were chosen by multi-stage stratified random cluster sampling. First, Xianju and Anji were selected as two representative mountainous counties, because the hilly/mountainous region in Xianju and Anji accounts for 80% and 70% of the territory, respectively. Next, towns in each county were ranked into three tiers using the economic development index, one town was randomly selected at each tier. Lastly, three villages, each of which represented one of three income tiers, were selected from the selected three towns. A total of 18 villages were randomly selected from two included counties. All residents in each selected village mandated to complete yearly physical check-up at local health centers, which is organized by local health department. This annual physical check-up is part of public health service that aims to identify any diseases at early stage. The records indicate that approximately 90% of residents participate in this annual physical check-up. Our staff invited all of those who presented themselves for physical check-up to participate in our study at the time our study conducted. Approximately 80% of population (9855/11898) in all the selected villages were enrolled.

A panel of 360 physicians from 18 primary care hospitals in the two selected counties were invited by the Department of Health, Zhejiang province, to conduct medical examinations, interviews, and laboratory tests on subjects who volunteered for this project. All local citizens and migrant workers who had settled in these cities for at least 3 months (i.e., a documented residence before July 1, 2012) were eligible for screening. The collected information consisted of demographics (sex, age, occupation, and nationality), residency status, medical history (e.g., blood transfusion, hepatitis B, allergies), and laboratory tests (HBsAg, anti-HBV surface antigen (anti-HBs), anti-HBV core antigen (anti-HBc), α-fetoprotein (AFP), alanine aminotransferase (ALT) assays, and liver ultrasound). This study was performed in accordance with the guidelines and regulations for the use of human subjects and was approved by the Ethics Committee of the First Affiliated Hospital of Zhejiang University College of Medicine. All adult participants provided informed consent, or informed consent was obtained from the legal guardian of the children. The data were handled anonymously.

### Serological testing

A 5-mL venous blood sample was collected from each participant, in accordance with strict hygiene and safety guidelines. The demographic information of the participants, including name, sex, age, and district, was recorded. Blood samples were kept in cool containers (4–10 °C) and delivered to Adicon Clinical Laboratories (Hangzhou, China), where sample processing and serological examinationwere performed on the same days. The HBV serological kits (ELISA) were purchased from Acon Biotech Co. (Hangzhou, China) for detection of HBsAg, HBsAb, and anti-HBc. In order to verify the test results, all samples were tested twice using the same kits. Only samples that were positive in both tests were considered positive. For the purpose of this analysis, HBsAg positivity was a marker for current HBV infection. Anti-HBc was indicative of previous exposure to HBV. Anti-HBs positivity without other HBV markers marked successful HBV vaccination. Individuals who tested negative for all HBV serological markers were categorized as having HBV exposure.

### Testing ALT and AFP levels

The serum ALT level was determined within 2 h of sample arrival by an Architect C8000 automated biochemical analyzer (Abbott Laboratories, Abbott Park, IL, USA). An abnormal ALT level was defined as greater than 38 IU/L, representing 2.25 standard deviations (SD) above the mean established for normal subjects. An elevated ALT level was verified by retesting the samples using the same kits. If a sample was positive in both tests, it was considered positive [13]. Community-based preliminary screening of hepatocellular carcinoma (HCC) was performed for HBsAg-positive subjects who were older than 30 years old. Both AFP (AFP EIA Test Kit, BoSai, Zhengzhou, China) and ultrasonography were used for HCC screening. Liver ultrasonography was conducted by technicians from the Imaging Department of each community health center. Based on a positive AFP test and a focal mass indicated by ultrasonography, a tentative diagnosis of HCC was suggested.

### Data analysis

The data were analyzed using SPSS software, version 21.0 (SPSS, Inc., Chicago, IL, USA). For categorical variables, the chi-squared test was used to determine group differences. Statistical significance was indicated when *P* < 0.01. The HBsAg, HBs-Ab, and HBc-Ab carrier rates were standardized by sex and age.

By referencing the 2010 Zhejiang population data [14], 95% confidence intervals (CIs) were calculated for percentages of HBsAg-positive individuals in the whole population and subgroups stratified by age and gender.

## Results

### Characteristics of the study group

**Fig. 1:**
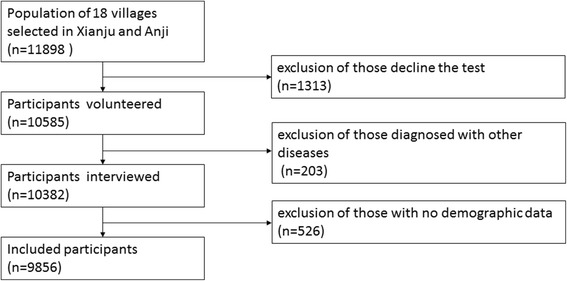
Flow chart of the selection process in the study populations

A total of 10,383 serum samples were tested. However, 528 participants (5.1%) were excluded because of no age or sex data. Thus, 9855 samples were included for final analyses (Fig. 1). The prevalence of HBV infection was standardized according to the age and sex distribution of all participants to allow direct comparisons of the studied populations. The demographic characteristics of the participant populations are shown in Table 1.

**Table 1:** Demographic data of the study populations

Region	Population	Participants interviewed	Included participants	Percentage of males (%)	Mean Age ± SD (years)
Xianju	342,700	6362	5844	43.9	53.8 ± 17.5
Anji	466,552	4021	4011	39.8	41.4 ± 8.2
Total	809,252	10,383	9855	42.2	49.0 ± 15.9

### Prevalence of HBV infection by age and sex

**Fig. 2:**
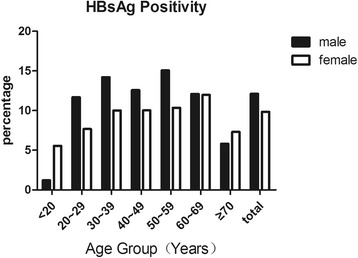
Age-specific prevalence of hepatitis B virus surface antigen (HBsAg)

In total, 1056 individuals were HBsAg positive (Table 2). The prevalence of HBsAg positivity, a marker of current infection, in this group was 9.5% (95% CI: 8.6–10.4). The HBV infection rate gradually increased from 3.23% in those <20 years old to a peak of 12.40% in those 50–59 years old, but then it declined to 6.6% in the group older than 70 years old. There was a significant difference in the prevalence of HBsAg between male (11.9%) and female participants (9.8%) (*P* < 0.01). Alarmingly, HBV infection in women of childbearing age remains high (5.56% in those <20 years old, 7.67% in those 20–29 years old, and 10% in those 30–39 years old) (Fig. 2).

**Table 2:** Prevalence of hepatitis B virus markers, stratified by age group

Age	Only anti-HBs		HBsAg		Anti-HBc		Anti-HBs	
	No. positive/no. tested	Percent	No. positive/no. tested	Percent	No. positive/no. tested	Percent	No. positive/no. tested	Percent
<20	75/155	48.4	5/155	3.2	22/155	14.2	85/155	54.8
20–29	131/562	23.3	51/562	9.1	180/562	32.0	224/562	39.9
30–39	406/1551	26.2	180/1551	11.6	641/1551	41.3	738/1551	47.6
40–49	1047/3385	30.9	374/3385	11.0	1340/3385	39.6	1671/3385	49.4
50–59	670/1839	36.4	228/1839	12.4	814/1839	44.3	1062/1839	57.7
60–69	437/1147	38.1	138/1147	12.0	531/1147	46.3	702/1147	61.2
>70	455/1216	37.4	80/1216	6.6	568/1216	46.7	773/1216	63.6
total	3221/9855	33.9	1056/9855	9.5	4096/9855	35.1	5255/9855	51.0

The positive percentages were standardized by age

**Fig. 3:**
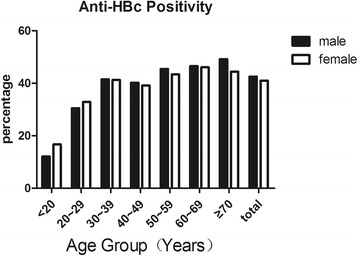
Age-specific prevalence of anti-HBV core antibody (anti-HBc)

**Fig. 4:**
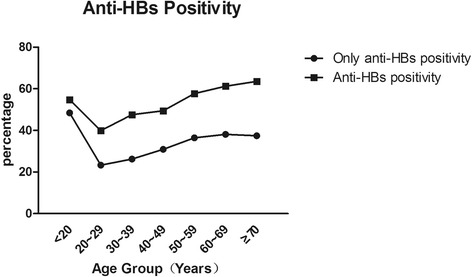
Age-specific prevalence of anti-hepatitis B surface antigen (anti-HBs) antibody positivity with or without other HBV markers

The prevalence of anti-HBc, a marker of HBV exposure, was 35.1% (95% CI: 33.4–36.8), and no significant variation was noted between genders. HBV exposure increased with age in both sexes, and it reached a peak in the group older than 70 years old, indicating that exposure to HBV had occurred in every age group (Fig. 3). The prevalence of anti-HBs increased from 39.9% to 57.7% in those aged 20–59 years old and to more than 60% among those >60 years old (Fig. 4). Having only anti-HBs positivity without other HBV markers was found in approximately 33.9% of the participants (95% CI: 31.5–36.3) (Table 2).

### Difference in prevalence of HBV infection between the two mountainous regions

As shown in Table 3, the prevalence of HBV infection was calculated according to gender in each region. The total prevalence was calculated through normalization with the urban population in addition to the sex ratio. The prevalence of HBsAg was significantly higher (*P* > 0.01) in the Xianju region (12.6%; 95% CI: 10.9–13.8) than in the Anji area (8.6%; 95% CI: 5.1–9.1). The HBsAg prevalence was higher in males than in females (*P* < 0.01) in both locations. The anti-HBc prevalence in Xianju (45.7%) was significantly higher than that in Anji (35.9%; *P* < 0.01), but there was no significant difference in anti-HBc positivity between males and females in either region. The anti-HBs prevalence differed significantly between Xianju (61.2%) and Anji (41.8%). The prevalence of vaccination-induced immunity (only anti-HBs positivity) was also significantly higher (P < 0.01) in Xianju (Table 3).

**Table 3:** Prevalence of hepatitis B virus markers, stratified by region

Region	Sex	Anti-HBs		HBsAg		Anti-HBc		Only anti-HBs	
		No. positive/total	Percent	No. positive/total	Percent	No. positive/total	Percent	No. positive/total	Percent
Xianju	Male	1583/2585	61.2	352/2585	13.6	1205/2585	46.6	1010/2585	39.1
	Female	2019/3301	61.2	383/3301	11.6	1475/3301	44.7	1227/3301	37.2
	Total	3602/5886	61.2	735/5886	12.6	2680/5886	45.7	2237/5886	38.1
Anji	Male	673/1595	42.2	154/1595	9.7	572/1595	35.9	422/1595	26.5
	Female	1002/2416	41.5	180/2416	7.5	867/2416	35.9	573/2416	23.7
	Total	1675/4011	41.8	334/4011	8.6	1439/4011	35.9	995/4011	25.1

Percentages of HBV markers were standardized by gender

### HCC screening in the HBsAg-positive group and ALT levels

**Fig. 5:**
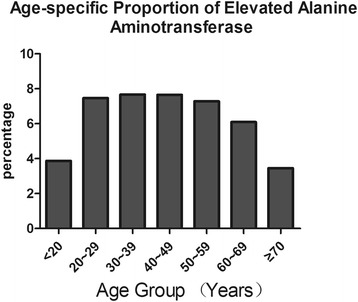
Age-specific percentages of elevated alanine aminotransferase (ALT) levels

Eight HBsAg-positive participants (6 men and 2 women, 0.11%), who were >40 years old, were AFP positive. However, no liver tumors were detected by ultrasonography. The mean (± SD) ALT level was 18.8 ± 7.4 IU/L, and the proportion of participants with an elevated ALT level (>38 IU/L) was 6.8%. The proportion of patients with an abnormal ALT level was much higher in the HBsAg-positive group (15.3%), compared with the HBsAg-negative group (6.3%) (*P* ≤ 0.01) (Table 4). The HBsAg-positive group had a significantly higher ALT level (25.32 ± 23.67 IU/L), compared with the HBsAg-negative group (18.05 ± 13.06 IU/L). Among all participants, the percentage of participants with an abnormal ALT level was significantly higher in males (9.55%) than in females (6.61%) (*P* ≤ 0.01). The average ALT level in males (21.40 ± 16.67 IU/L) was higher than that in females (16.95 ± 12.84 IU/L). Additionally, the proportion of patients with an abnormal ALT level markedly decreased with age, from 7.67% in the group aged 30–39 years old to 3.45% in the group of subjects >70 years old (*P* ≤ 0.01) (Fig. 5).

**Table 4:** Percentages of elevated ALT in HBsAg-positive and HBsAg-negative groups

	HBsAg-positive						HBsAg-negative					
	<38 UI/L			>38 UI/L			<38 UI/L			>38 UI/L		
	No. positive/Mean			No. positive/Mean			No. positive/Mean			No. positive/Mean		
Serum		ALT ± SD	%		ALT ± SD	%		ALT ± SD	%		ALT ± SD	%
ALT	no. test			no. test			no. test			no. test		
Male	398/499	20.19	79.8	101/499	65.62	20.2	3364/3660	17.34	91.9	296/3660	54.63	8.1
		7.50			50.84			7.66			20.41	
Female	501/557	17.65	89.9	56/557	57.86	10.1	4920/5139	14.59	95.7	219/5139	57.85	4.3
		7.06			20.02			6.34			30.78	
Total	899/1056	18.76	84.7	157/1056	62.85	15.3	8284/8799	16.69	93.7	515/8799	56.00	6.3
		7.35			42.57			6.79			25.37

## Discussion

We conducted a new epidemiological study of HBV infection in two mountainous regions of Zhejiang province and provided a fresh and interesting peek into the current status of HBV infection in China. We found that HBsAg positivity in the general population of the two study regions remains considerably high and that it increased with age. We also found that HBV infection in women of childbearing age is still at a high level (up to 10%). Finally, we found that the prevalence of HBV infection depends on the location, despite a similar landscape of the two areas.

The positive HBsAg rate in the mountainous areas of Zhejiang province decreased to 10.19% in 2011, from 15.94% in 1993 [9, 11]. The current study shows a chronic HBV infection rate of 9.5% in two mountainous areas of Zhejiang, which is lower than that reported previously but still higher than average national or provincial levels.

Maternal-neonatal HBV transmission is the main route for the spread of HBV infection in China. In Zhejiang province, the EPI vaccination program is considered a success. Since the program was initiated in 1992, HBV infection in the participants who were younger than 20 years old was reduced to 3.2%, compared to a HBV infection rate of 9.5% in the total studied population. The success of universal HBV immunization of infants directly contributes to the decline in the HBV infection prevalence in China. However, the achieved success is not good enough because a portion of participants younger than 20 years old are infected with HBV. There are two possible reasons for the incomplete blockade of HBV transmission. One is HBV immunization coverage. Chen Xuejun et al. have reported that the average HBV vaccination coverage rate in Zhejiang was more than 95%, but not 100% [14]. A gap in the coverage can partially explain the data. The other reason could be related to the response rate to HBV immunization. As shown by our data, only anti-HBs positivity in the group younger than 20 years old was only around 50%. Assuming that 95% of them were immunized with the HBV vaccine, nearly 40% of immunized participants did not have detectable anti-HBs antibody, suggesting that the detectable anti-HBs antibody level has waned out or the response of the anti-HBs antibody to the HBV vaccine is unexpectedly lower than the reported 95% [14]. In a recent study, nearly 22% of HBV-vaccinated infants were negative for HBV-neutralizing antibody when they were tested at the age of one year (6 months after the last vaccine injection), and they had to be revaccinated, suggesting that the HBV-neutralizing antibody levels quickly declined or were initially lower than 10 mIU/mL [15]. Our results raise a serious question about the effectiveness of the current HBV vaccine and vaccination schedule. Clearly, our findings need to be verified in larger populations across different geographic locations.

Another finding that surprises us is that chronic HBV infection in women younger than 20 years old was more than 5%. This result could be statistically skewed because of the relatively smaller number of participants in the group younger than 20 years old. In other age groups, the detected HBsAg positivity in females was generally lower than that in males. Despite relatively lower HBsAg positivity in females, the prevalence of chronic HBV infection among women of reproductive age (20–40 years old) is 9.36%, which is still a very high level. Chronic HBV-infected women of reproductive age represent an important source for HBV transmission. It has been reported that the HBV vaccination rate in mountainous regions was significantly lower than that in plains areas [16]. Incomplete coverage of HBV immunization and a lower response rate of anti-HBs antibody in immunized children will contribute to perinatal HBV infection, the majority of whom will become chronically infected. Our results reflect a severe challenge that we are facing in completely blocking the vertical transmission of HBV. Government agencies ought to expand HBV immunization coverage and strengthen the screening test for HBsAg among pregnant women to increase the HBV interruption rate of perinatal transmission [17–19]. Additional HBV immunization drugs that are more effective than the currently available hepatitis B immunoglobulin and HBV vaccine are needed to completely block the vertical transmission of HBV.

Several reasons have been suggested for the relatively high chronic HBV infection in the hilly region: first, the economy in the mountainous region is at a lower level compared to other regions that are easily accessible by transportation. Sun et al. have found that the positive rate of HBsAg in less-developed regions was significantly higher than that in developed areas [20]; second, the population living in the mountainous villages may not have sufficient awareness of HBV infection and lack protection against HBV transmission in daily life, which may have caused the spread of hepatitis B infection [21, 22]; third, poor hygiene in the hilly region is also a risk factor for HBV infection B [23]; finally, the primitive lifestyle of those who live in hilly regions makes HBV spread easily [24].

The results of this study showed that the HBcAb-positive rate increased with age, which is consistent with the results of an earlier survey [25]. Thus, horizontal HBV transmission may occur more frequently than realized previously.

Interestingly, the distribution of anti-HBc by age was not different between the two genders (Fig. 2), while the distribution of HBsAg by age did not follow the same pattern (Fig. 1): The HBs-Ag positive frequency in male adults was much greater than that of female adults. This finding indicates that while exposure to HBV transmission among male and female subjects was similar, a higher portion of the HBV-exposed male adults became chronically infected in this cohort. This observation was consistent with the pattern described by a long-term study of HBV infection in Fujian [26].

The current survey showed that the positivity rates of HBsAg and anti-HBc in the Xianju area were significantly higher than those in the Anji region (Table 3). Anji is located in the Yangtze River delta and has a more developed economy than Xianju. In addition, the prosperous tourist industry in Xianju may have facilitated the spread of HBV infection [27]. Though both areas were included in the EPI in 1992, which initially only covered infants, Xianju started implementing free HBV immunization for all residents including infants and adults in 2010 to expand immunization. These facts may explain the difference in the prevalence of only anti-HBs detection between the two regions.

The association between abnormal ALT levels and HBsAg status was also indicated in the current study. The HBsAg-positive group had higher levels of ALT, compared with the HBsAg-negative group. Furthermore, abnormal ALT levels were significantly more frequent among HBsAg carriers than those who were not carriers.

HCC is the fifth most common malignancy and the third leading cause of cancer-related deaths worldwide. Approximately 85% of HCC cases are borne in developing countries, especially in East and Southeast Asia and sub-Saharan Africa, where a major risk factor is chronic HBV infection [28, 29]. The prevalence of abnormal AFP levels among HBsAg-positive individuals aged >30 years old was 0.8% in this group, greater than the recently reported 0.13% found in the general population of the same age range in Zhejiang province [30]. Although no HCC cases were identified among the AFP-positive individuals by the ultrasound examination, an elevated APF level warrants close monitoring.

We acknowledge several limitations in this study. First, as a community-based epidemiology study, we did not collect information on genotype, IgM antibody, and participants’ vaccine history, which may better inform the HBV epidemiological characteristics in study areas. Second, the exclusion of those who missed the information of age and gender from our analysis may have potential impact on overall prevalence estimate. Moreover, the exclusion of those who were diagnosed with hepatitis C, hepatitis D, other liver diseases, HIV, immune-suppression disorders, or those who were undergoing antiviral therapy may have underestimated the HBV prevalence because they were probably at greater risk of being infected with hepatitis B. Finally, we did not collect data on risk factors of these populations, including homosexual activity, intravenous drug use, transfusion, and shared medical equipment (e.g., dialysis and dental procedures).

## Conclusions

In conclusion, this community-based study of HBV infection in hilly/mountainous regions in China describes the current status of HBV infection in China. We found that after more than 20 years of the EPI, HBsAg positivity in the general population of the two study regions remains considerably high at 9.5%, which is significantly higher than the national level. We also found that HBV infection in women of childbearing age is still at a high level (up to 10%). Finally, we found that the prevalence of HBV infection depends on the location, despite a similar mountainous landscape of the two areas. Our data serve as a reminder that it is a long journey to block HBV transmission completely through HBV vaccination alone in China and that a more effective treatment that can clear chronic HBV infection is required to lower the prevalence of HBV infection in China.

## Abbreviations

AFP: α-fetoprotein
ALT: Aminotransferase
anti-HBc: Anti-HBV core antigen
anti-HBs: Anti-HBV surface antigen
HBsAg: Hepatitis B virus surface antigen
HBV: Hepatitis B virus
